# Spatially Reciprocal Inhibition of Inhibition within a Stimulus Selection Network in the Avian Midbrain

**DOI:** 10.1371/journal.pone.0085865

**Published:** 2014-01-21

**Authors:** C. Alex Goddard, Shreesh P. Mysore, Astra S. Bryant, John R. Huguenard, Eric I. Knudsen

**Affiliations:** 1 Department of Neurobiology, Stanford University, Stanford, California, United States of America; 2 Department of Neurology, Stanford Medical School, Stanford, California, United States of America; CSIC-Univ Miguel Hernandez, Spain

## Abstract

Reciprocal inhibition between inhibitory projection neurons has been proposed as the most efficient circuit motif to achieve the flexible selection of one stimulus among competing alternatives. However, whether such a motif exists in networks that mediate selection is unclear. Here, we study the connectivity within the nucleus isthmi pars magnocellularis (Imc), a GABAergic nucleus that mediates competitive selection in the midbrain stimulus selection network. Using laser photostimulation of caged glutamate, we find that feedback inhibitory connectivity is global within the Imc. Unlike typical lateral inhibition in other circuits, intra-Imc inhibition remains functionally powerful over long distances. Anatomically, we observed long-range axonal projections and retrograde somatic labeling from focal injections of bi-directional tracers in the Imc, consistent with spatial reciprocity of intra-Imc inhibition. Together, the data indicate that spatially reciprocal inhibition of inhibition occurs throughout the Imc. Thus, the midbrain selection circuit possesses the most efficient circuit motif possible for fast, reliable, and flexible selection.

## Introduction

The ability to select one among several, competing alternatives is crucial for an animal's survival. Competitive selection is observed in a range of processes such as perception, attention, and decision-making. Frequently, this selection must be flexible, with the selection boundary shifting as the set of alternatives changes. For instance, if an animal must select the faster of two vibrational stimuli, it must be able to do so regardless of the absolute vibration frequencies of the stimuli [1].

**Figure 1:**
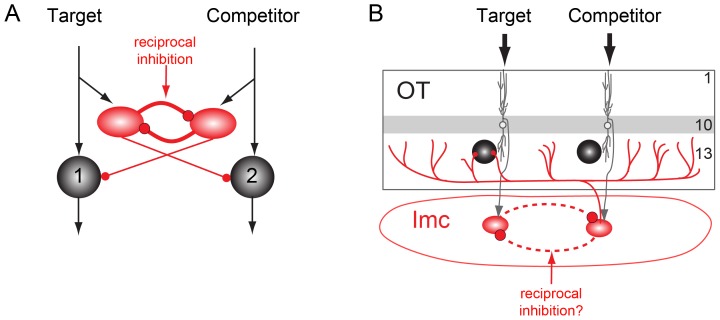
Reciprocal inhibition of inhibition in the midbrain selection network. A) Computational model: Schematic of a lateral inhibitory circuit with long-range projections from the inhibitory units (red ovals) to the excitatory output units (black circles). Black arrows indicate excitatory connections, red circles represent inhibitory connections. One channel (1) represents a target stimulus; the other (2) represents a competitor stimulus. Reciprocal feedback inhibition between inhibitory units is depicted with solid lines. Adapted from [2]. B) Anatomy: Schematic of midbrain selection network. Neurons in layer 10 of the OT (white circles, grey dendrites) send topographic projections to Imc neurons (red ovals). Imc neurons send widespread GABAergic projections to neurons in the intermediate/deep layers of the OT (black circles). Putative reciprocal inhibition between spatial channels is depicted with dashed lines.

A recent computational model demonstrated that such flexible selection requires feedback inhibition among the competing channels of information [2]. One particular implementation, reciprocal inhibition of lateral inhibition (Fig. 1A), was identified as being the simplest, structurally, and it outperformed other implementations in terms of speed and reliability of selection. Given this theoretical finding, we sought to determine whether the brain employs this highly efficient implementation in networks that participate in flexible selection.

An excellent site to investigate this question is a network in the vertebrate midbrain that plays a critical role in stimulus selection for gaze and attention [3]. This network flexibly signals the strongest of all competing stimuli regardless of their absolute strengths [4], [5]. In birds, this network includes the optic tectum (OT; superior colliculus in mammals) and a specialized GABAergic nucleus in the midbrain tegmentum, called the nucleus isthmi pars magnocellularis (Imc)[6]. The Imc receives topographic input from the OT and sends back inhibitory output globally to the OT space map (Fig. 1B) [7]. According to the computational model, reciprocal inhibitory connectivity within the Imc could explain the flexible selection of the strongest stimulus observed in the OT. This pattern of connectivity would be established by intranuclear, long-range inhibition between all spatial locations within the Imc space map.

This study explores the nature and spatial pattern of connectivity within the Imc. Using laser-scanning photostimulation with caged glutamate in an acute slice preparation of the avian midbrain selection network, we demonstrate that the Imc does, indeed, contain this specialized circuit motif of monosynaptic, long-range inhibition. We demonstrate that this motif functions globally across the Imc space map: unlike typical feedforward inhibition, the strength of inhibition does not decline with distance. Finally, we report extensive, intrinsic anatomical projections within the Imc that can support this spatially reciprocal, global inhibition.

## Results

### Photoactivation of Imc neurons

**Figure 2:**
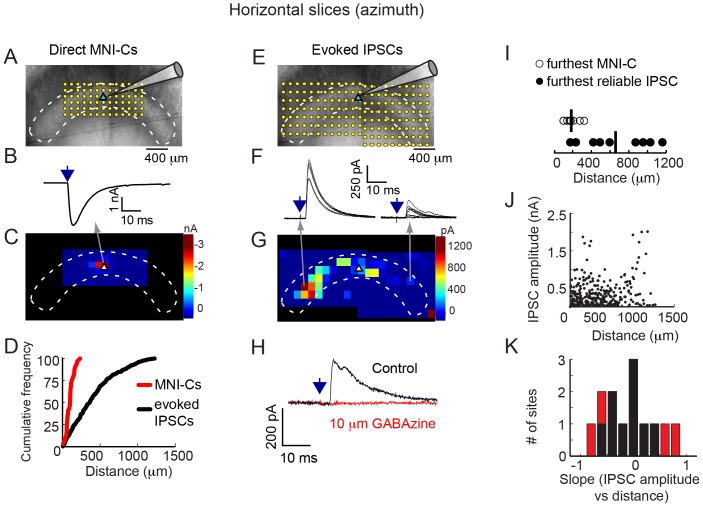
Intra-Imc inhibition in the horizontal plane. A) Image montage of the Imc in the horizontal plane. White dashed lines outline the Imc. The blue triangle (near the center of Imc) indicates the location of the recorded neuron. The grid of yellow dots represents locations of laser photostimulation across the Imc for assessing direct MNI-Cs. B) Example trace showing a single, direct MNI-C, recorded at −60 mV, evoked from the location indicated in the map in Fig. 2C. Arrow indicates time of photostimulation. C) Map of direct MNI-Cs for the neuron depicted in Fig. 2A. MNI-Cs were observed only at sites near the recorded neuron. White triangle represents location of the recorded neuron. D) Cumulative frequency plot of MNI-Cs (red, 7 maps from 7 neurons) and IPSCs (black, 13 maps from 9 neurons) recorded in the horizontal plane. Median distance of the distributions (followed by 25^th^ and 75^th^ percentiles) were: MNI-C: 97 µm (57–111 µm), IPSC: 362 µm (167–580 µm). E) Image montage of the Imc in the horizontal plane, showing two maps acquired on either side of the recorded neuron. Same conventions as in Fig. 2A. F) Example traces showing multiple, reliable photostimulation-evoked IPSCs from the two locations indicated in the map in Fig. 2G. Left traces are evoked by photostimulation at a site 1 mm away from recorded neuron; right spot by photostimulation at a site 880 µm away. Arrow indicates time of photostimulation. G) Map of evoked IPSCs for the neuron depicted in Fig. 2A and E. IPSCs were evoked from either side of the recorded neuron. H) IPSCs evoked by photostimulation were eliminated by 10 µM GABAzine. Arrow indicates time of photostimulation. I) Distances of the furthest MNI-C or furthest reliable IPSC for Imc neurons recorded in the horizontal plane. Mean distance (horizontal line) of MNI-C: 208 µm (n = 7), of IPSC: 680 µm (n = 9 neurons). J) Comparison of photostimulation-evoked IPSC magnitude to photostimulation site distance from recorded cell. Large amplitude IPSCs could be evoked from sites proximal to or distal to the recorded neuron. IPSCs from all recordings (13 maps from 9 neurons) are shown. K) Histogram of slopes from regressions comparing IPSC amplitude to photostimulation distance for individual maps. Only 4 of 13 regressions were significant (p<0.05; red bars), 2 with slope <0 and 2 with slope >0.

To test if intranuclear inhibition is present within the Imc (Fig. 1), we first tested for the presence of direct inhibitory connectivity within the Imc. We prepared 300 µm acute slices of the chick midbrain cut in the horizontal plane, which encodes spatial azimuth. We recorded from Imc neurons in whole-cell voltage clamp mode while simultaneously using laser-scanning photostimulation of MNI-glutamate to focally excite neurons at various locations across the extent of the Imc (Fig. 2A). We delivered 100–200 µs pulses of 355 nm light, using a grid pattern with a spacing of 75–125 µm between neighboring sites (Fig. 2A).

We first characterized the efficacy of photostimulation by recording direct, excitatory MNI-mediated currents (MNI-C) by clamping the neuron's membrane potential at −60 mV (Fig. 2B). MNI-Cs occurred at short latencies (1–4 ms) and were large (peak amplitude: 2364±942 pA, n = 7 neurons), consistent with levels of current required to evoke action potentials from Imc neurons (median rheobase  = 1110 pA, n = 8 neurons). MNI-Cs were evoked by photostimulation only near the recorded neuron, within ∼200 µm of the recording site (Fig. 2C and 2D, red curve). When spiking activity was recorded in cell-attached mode, we found that photostimulation within 200 µm of the recorded Imc neuron generated a single, short latency spike (mean furthest distance of evoked spike  = 146±33 µm; 1.1±0.2 spikes per photostimulation; mean latency  = 3.2±0.9 ms, n = 5 neurons). Thus, we established photostimulation conditions that reliably evoked single spike responses from Imc neurons when applied in the immediate vicinity of the recorded neuron.

### Photoactivation of Imc neurons drives IPSCs

We next tested whether activating neurons at various locations across the Imc with photostimulation induced inhibition in a recorded Imc neuron (Fig. 2E). We isolated inhibitory postsynaptic currents (IPSCs) by holding the neuron's membrane potential at 0 mV. IPSCs were detected at latencies of 5–20 ms. In contrast to the excitatory MNI-Cs, we found that IPSCs were evoked by photostimulation across the extent of the Imc, at more distant sites than MNI-Cs (p<0.001, comparing distances of IPSCs to MNI-Cs, Mann-Whitney U-test, n = 13 maps from 9 neurons). As the horizontal dimension of the Imc can extend >4 mm, some sites required multiple, adjacent scanning sessions to test for synaptic connectivity on either side of the recorded neuron (Fig. 2F–G). These IPSCs were abolished by bath application of the GABA_A_ antagonist GABAzine (Fig. 2H). Whereas some of the IPSCs (29%) were evoked by photostimulation at locations near the recorded neuron (<200 µm, the extent of MNI-C), the majority of IPSCs (71%) were evoked by photostimulation at distant locations (>200 µm; Figs 2D, black curve). Across the population of recorded neurons, IPSCs were evoked by photostimulation on either side of the neuron and as far away as 1225 µm (Fig. 2D, black line).

To assess the prevalence of spontaneous IPSCs (“false positives”) in our sample, we counted the number of IPSCs detected from photostimulation sites beyond the anatomical boundaries of Imc. These sites were located in fiber tracts or in a neighboring, non-Imc projecting, cholinergic nucleus. IPSCs were detected for 40 out of 329 such sites (n = 13 maps from 9 neurons), a contamination rate of ∼12%. Thus, a majority of the recorded IPSCs corresponded to input from photostimulated Imc neurons.

To verify that IPSCs could be evoked at long-ranges from the recorded neuron, we delivered photostimulation repeatedly at a subset of photostimulation sites. Sites at which IPSCs were detected in response to >50% of the photostimulation pulses were referred to as being “reliable” sites” (e.g., (Fig. 2F). At these sites, an IPSC was observed in response to, on average, 95.9±11.3% of the photostimulation pulses delivered. Consistent with the results from the initial survey, IPSCs at reliable sites were located at distances further from the recorded neuron than those that evoked MNI-Cs (Fig. 2I, p<0.005, Mann-Whitney U-test, n = 9 neurons), with the farthest reliable IPSC site being 1160 µm away.

Although inhibition could potentially be evoked by photostimulation at sites anywhere across the Imc, the spatial pattern and density of effective sites varied across different slice experiments. This observation probably reflects the likelihood that long-distance axons were severed during the preparation of the acute slices. Despite this limitation, large magnitude IPSCs were evoked by photostimulation at sites over 1 mm away from recorded neurons. Importantly, the strength of the evoked IPSCs did not decrease with increasing distance. Large magnitude IPSCs were found distally and proximally to the recorded neuron (Fig. 2G, J), with the largest amplitude IPSCs averaging close to 1 nA (975±620 pA, n = 13 maps from 9 neurons). We compared IPSC amplitude with photostimulation distance using linear regression: across all experiments, there was no relationship between these measures (Fig. 2J, slope  = 0.02 pA/mm, p>0.99, n = 13); for individual experiments, we found significant (p<0.05) relationships for 4 out of 13 neurons: 2 with slopes>0 and 2 with slopes<0 (Fig. 2K). These data indicate, therefore, that the strength of lateral inhibition remains essentially constant across the Imc in the horizontal plane. Thus, the Imc contains neurons that send strong lateral inhibition throughout the nucleus.

**Figure 3:**
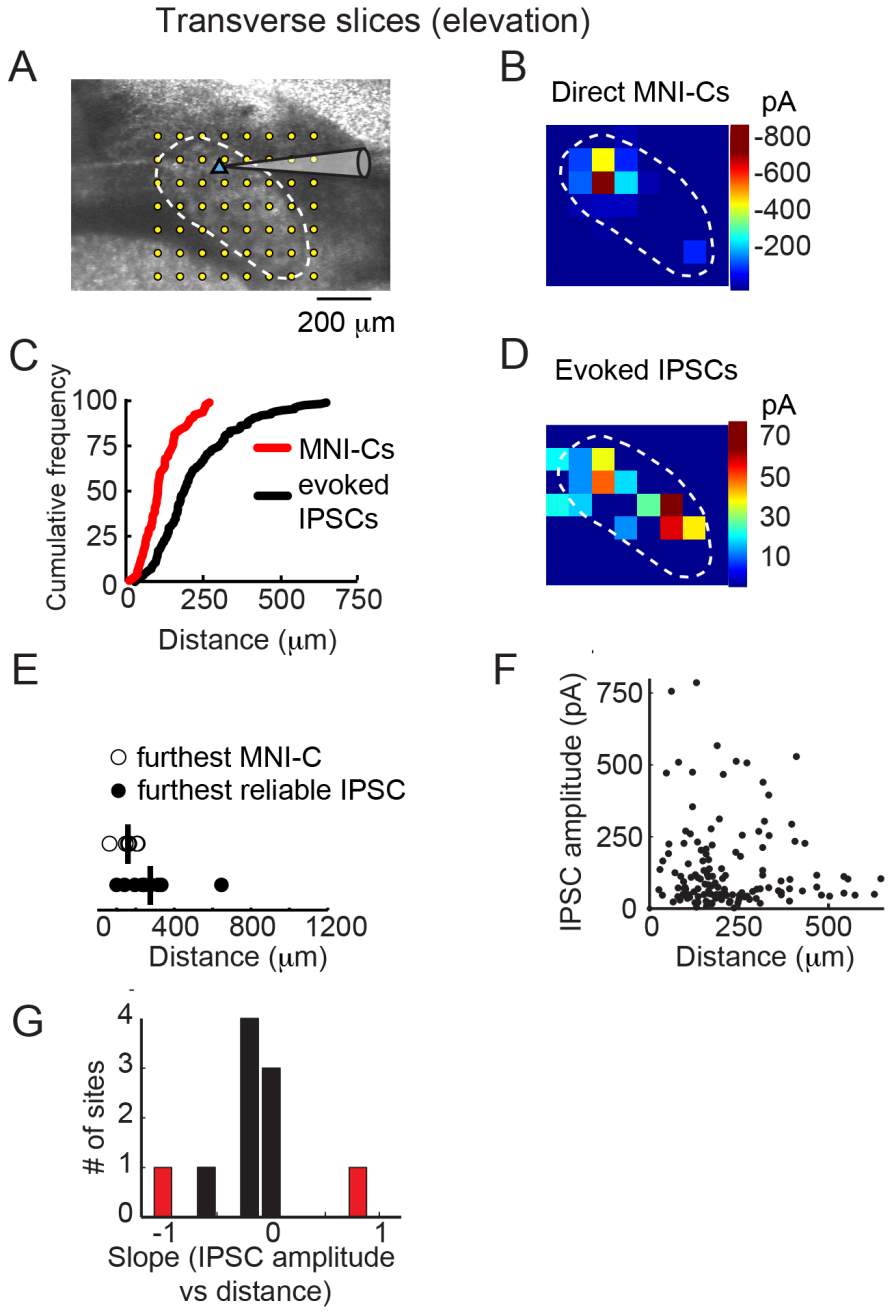
Intra-Imc inhibition in the transverse plane. A) Image montage of the Imc in the transverse plane. Conventions as in Fig. 2A. B) Map of direct MNI-Cs for the neuron depicted in Fig. 3A. MNI-Cs were evoked only from sites near the recorded neuron. Conventions as in Fig. 2C. C) Cumulative frequency plot of MNI-Cs (red, 7 maps from 7 neurons) and IPSCs (black, 10 maps from 10 neurons) recorded in the horizontal plane. Median distance of the distributions (followed by 25^th^ and 75^th^ percentiles) were: MNI-C: 121 µm (70–202 µm), IPSC: 184 µm (127–293 µm). D) Map of evoked IPSCs for the neuron depicted in Fig. 3A. IPSCs were evoked from sites throughout the Imc. E) Distances of the furthest MNI-C or furthest reliable IPSC for Imc neurons recorded in the transverse plane. Mean distance (horizontal line) of MNI-C: 167 µm (n = 7), of IPSC: 276 µm (n = 10). p<0.05, Mann-Whitney U-test. F) Comparison of photostimulation-evoked IPSC magnitude to photostimulation site distance from recorded cell. Large amplitude IPSCs could be evoked from sites proximal to or distal to the recorded neuron. IPSCs from all recordings are shown. G) Histogram of slopes from regressions comparing IPSC amplitude with photostimulation distance for individual maps. Only 2 of 10 regressions were significant (p<0.05; red bars), 1 with slope <0 and 1 with slope >0.

We also tested for long-range inhibition in Imc slices cut in the transverse plane, which encodes spatial elevation (Fig. 3). The representation of elevation is more compressed than that of azimuth: the longest dimension of the Imc measures only 600–800 µm in the transverse plane compared with ∼4 mm in the horizontal plane. As in horizontal slices, direct MNI-Cs were evoked in transverse slices by photostimulation only at sites close to the recorded neuron, <200 µm away (Fig. 3A–C). Also, IPSCs were evoked by photostimulation at more distant sites, up to 646 µm from the recorded neuron, near the edges of the nucleus (Fig. 3C–D, p<0.001 comparing distribution of distances of IPSCs to MNI-Cs, Mann-Whitney U-test, n = 10 maps from 10 neurons). An analysis of IPSCs evoked from reliable sites supported this observation (Fig. 3E). Across all experiments, there was no consistent relationship between IPSC amplitude and distance from the recorded neuron (Fig. 3F; slope  = −0.1 pA/mm, p>0.40); for individual experiments, we found significant (p<0.05) relationships for 2 out of 10 neurons: 1 with slope>0 and 1 with slope <0 (Fig. 3G). These data indicate that, as in the horizontal plane, the strength of lateral inhibition remains essentially constant across the nucleus in the vertical plane.

### Extensive anatomical projections within the Imc

The computational model demonstrated that two components are necessary for efficient, flexible selection: 1) intranuclear inhibition and 2) spatial reciprocity. Having demonstrated the presence of intranuclear inhibition, we used anatomical tracing methods to test for spatial reciprocity. We used two different anatomical methods to visualize intra-Imc cytoarchitecture, both involved making small injections of bi-directional tracers into the Imc.

**Figure 4:**
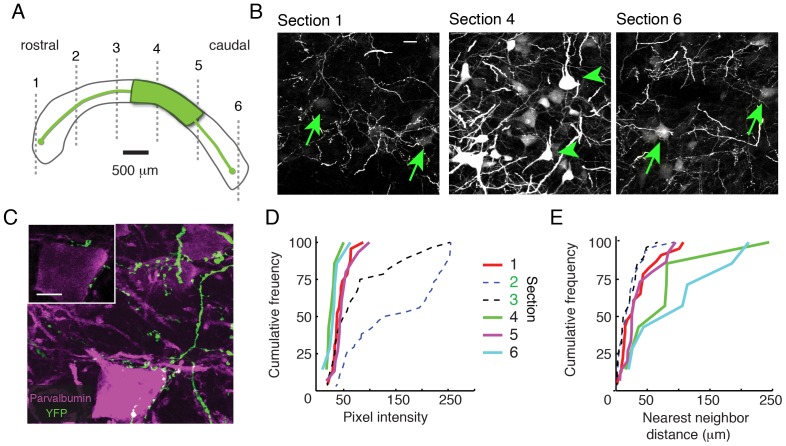
Long-range axonal projections and retrograde labeling within the Imc *in vivo*. A) Schematic showing the location of injection of A3V expressing eGFP (green) in the horizontal plane. Grey, dashed lines represent the locations of the transverse sections shown in the following panels. B) eGFP-labeled axons and somata in transverse sections across the rostrocaudal extent of the Imc. Green arrows indicate retrogradely labeled somata observed at the rostral and caudal poles of the Imc (Sections 1 and 6). Green arrowheads indicate directly labeled somata observed only near the injection site (Section 4). C) Maximum projection confocal image of eGFP-labeled boutons (green) surrounding a parvalbumin-immunostained Imc somata (purple). *Inset* (upper left): single plane confocal image showing close apposition of an eGFP-expressing axon and the Imc soma shown in the full image (bottom right). D) Quantification of pixel intensity for somata at various locations across the rostrocaudal extent of the Imc. Each line represents distribution from a particular section in the transverse plane. A majority of neurons near the injection site (dashed lines) exhibited high eGFP expression, as indicated by high pixel intensity, while those at the distal ends consistently exhibited lower intensity (solid lines). E) Quantification of density of labeled somata at various locations across the rostrocaudal extent of the Imc. The distance of the nearest labeled neighbor was determined for each neuron in a field of view. Neurons near the injection site (dashed lines) had close near-neighbors, suggesting a dense distribution of labeled neurons. Neurons at the distal ends (solid lines) had more distant near-neighbors, suggesting a sparse distribution of labeled neurons.

In the first experiment, we studied patterns of anterograde and retrograde labeling following an injection of an Avian specific Adeno-Associated virus (A3V) expressing eGFP into the Imc *in vivo* (Fig. 4). The injection was made in the middle of the rostrocaudal extent of the Imc. We waited 19 days post-injection to permit high levels of expression and transport of eGFP, after which we prepared transverse sections of the midbrain for histology.

Following virus injection *in vivo*, anterograde axonal labeling and retrograde somatic labeling was abundant throughout the Imc. eGFP was observed near the injection site, which extended over the central ∼1.3 mm of the nucleus (Fig. 4A, 4B center). Outside of this zone, anterograde and retrograde labeling was remarkably uniform, a pattern of labeling distinct from conventional, topographically restricted, inhibitory connectivity [10]. eGFP-labeled axons, many with boutons, were observed throughout the Imc, all the way to the rostral and caudal poles of the nucleus (Fig. 4B, left and right). We immunostained some of these sections for parvalbumin, a protein expressed robustly by Imc neurons and a marker for inhibitory neurons in other brain regions [8]. Using confocal microscopy, we observed eGFP-labeled boutons in close apposition to parvalbumin-immunostained somata, suggesting that labeled axons were providing perisomatic input to Imc neurons (Fig. 4C). This pattern of long-range axonal labeling, from the center out to the distal regions of the Imc, suggests that intra-nuclear axonal projections are global.

The pattern of retrograde somatic labeling from the same injection was consistent with spatially reciprocal connectivity. In addition to labeled axons, we also found eGFP-expressing somata were widespread across a large extent of the nucleus. Two different classes of somatic labeling were observed. First, strongly labeled somata were found exclusively near the injection site, extending over ∼1.3 mm (Fig. 4B, middle panel; Fig. 4D, dashed lines). These strongly labeled somata tended to be densely clustered, with short distances between nearest-neighbors (Fig. 4E, dashed lines). The high expression of eGFP and dense clustering of these neurons is consistent with direct infection by virus diffusing locally from the injection site.

In comparison, weakly labeled somata were found throughout the remainder of the Imc (Fig. 4B, left and right panels; Fig. 4D, solid lines). These weakly labeled somata were distributed sparsely, with longer distances between nearest-neighbors (Fig. 4E, solid lines). The weak expression of eGFP, lack of clustering, and distance of these neurons from the injection site are all features consistent with the neurons expressing retrogradely transported virus [11]. This widespread pattern of retrogradely labeled somata is consistent with the functional pattern of inputs observed in the in vitro physiology experiments (Fig. 2G) and is roughly the inverse of the anatomical pattern of axonal projections. The relatively sparse clustering of retrogradely labeled somata is suggestive of spatial, rather than neuron-by-neuron reciprocity. In combination, these data provide strong support for spatially reciprocal, global inhibition.

We confirmed this finding with a second, independent approach. Focal injections of a bi-directional tracer (fluororuby) were made *in vitro*, in horizontal slices, which allowed for precise, visualized targeting of the Imc. For these experiments, the midbrain was sliced into thick, 500 µm sections to reduce the transection of long distance axons. Two experiments were done: in one, the injection was made near the middle of the Imc (reproducing the eGFP experiment), and in the other, the injection was made near the rostral pole of the nucleus.

**Figure 5:**
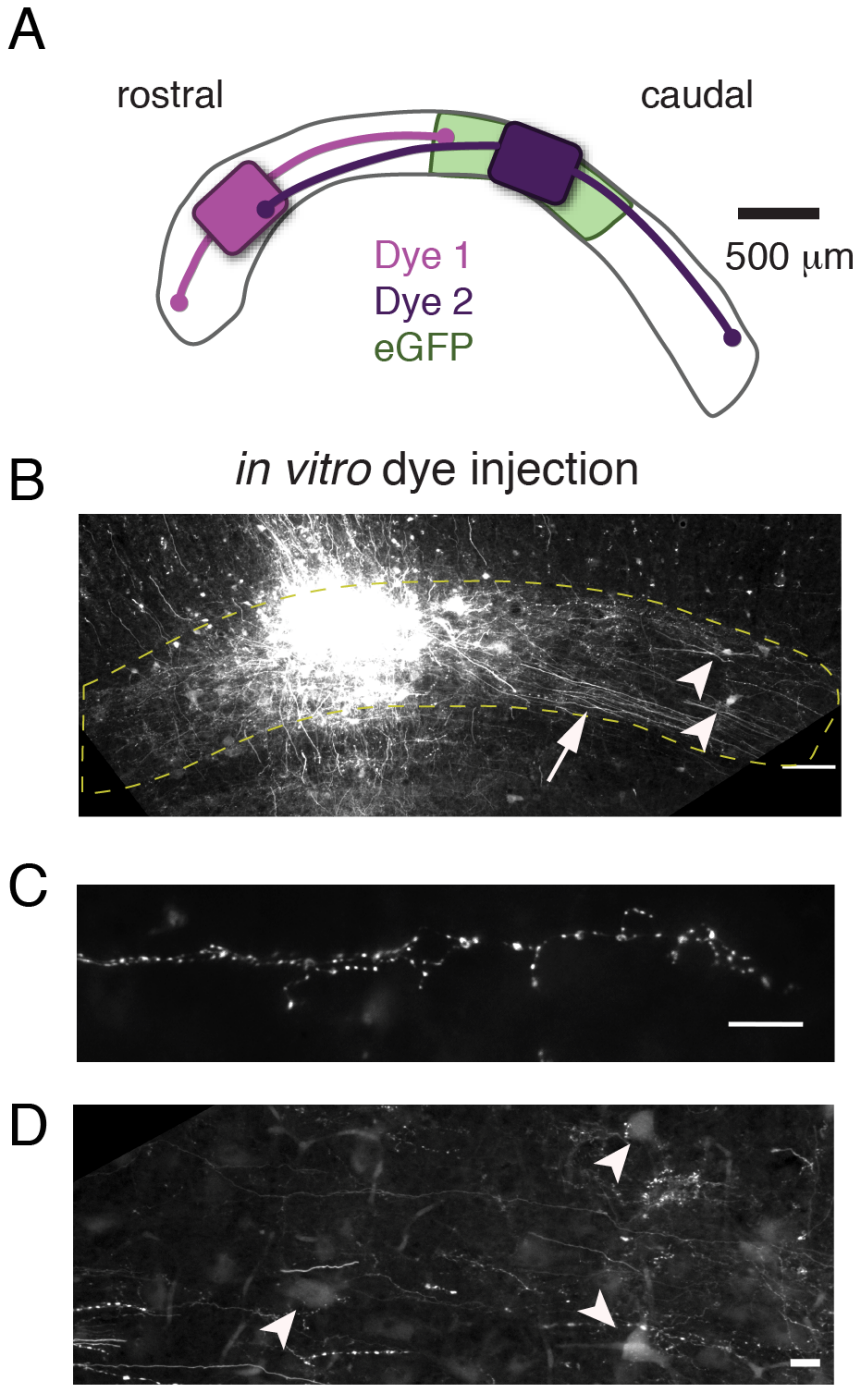
*In vitro* labeling of long-range axonal projections and somata in the horizontal plane. A) Schematic showing the locations of injections of fluororuby dye (purple and lavender) along with approximate distances of observed axonal projections (curved lines). Light green shading indicates location of eGFP injection shown in Fig. 4 for context; virus and fluororuby injections were not performed in the same tissue. B) Low power image of the Imc, cut in the horizontal plane, following an injection of fluororuby. Horizontally oriented axons (arrow) can be observed streaming from the injection site. Retrogradely labeled somata are apparent away from the injection site (arrowheads). Scale bar  = 100 µm. C) High power image of labeled axons, distant from the dye injection site. Boutons can be observed along the axon. Scale bar  = 10 µm. D) High power image of retrogradely labeled somata (arrowheads), distant from the dye injection site. Scale bar  = 10 µm.

Similar to the results from the viral injection *in vivo*, fluororuby injections *in vitro* revealed anterograde and retrograde labeling throughout large extents of the Imc (Fig. 5A, B). As predicted by the viral injection, numerous labeled axons extended long distances in both directions from the injection sites. These axons coursed within and parallel to the rostrocaudal axis of the nucleus, giving off branches with clusters of terminal boutons (Fig. 5C). In the same tissue, retrogradely labeled somata were scattered across large extents of the nucleus (Fig. 5D). Because of the likelihood of transected connections in these slice preparations, the spatial profile of labeling was not quantified. However, the qualitative projection patterns from these two experiments were consistent with all predictions from the *in vivo* labeling experiment (which was not subject to this confound), and supported the conclusion that inhibitory connectivity within the Imc is global and spatially reciprocal.

In summary, both the viral and tracer injections demonstrated globally projecting patterns of axons extending *from* the injection sites that we describe as “one location contacts many locations.” Simultaneously, we saw a mirror-image pattern of retrogradely labeled neurons projecting *to* the injection site that we describe as “many locations to one location.” These findings are in close agreement with the physiological results. Moreover, because these patterns were observed independently of where the injection or recording site was located along the rostrocaudal axis of the Imc, we conclude that spatially reciprocal inhibition is a fundamental property of the Imc.

## Discussion

This study demonstrates that a motif of intrinsic feedback connectivity, reciprocal inhibition of laterally-projecting inhibitory neurons, exists within the Imc, an inhibitory nucleus that is essential for competitive stimulus selection in the midbrain selection network [2], [6]. Our results build on previous studies, which presented anatomical evidence for GABAergic terminals and local axon collaterals within the Imc [7], [12]. Importantly, we establish the functionality of these collaterals, and their spatial reciprocity. Although this study did not explore the possibility of reciprocal inhibitory connections between pairs of neurons, it did demonstrate reciprocal inhibition between pairs of *spatial representations* within the Imc, as required by the computational model.

One caveat of the anatomical experiments is that fibers of passage may have been labeled by the viral and dye injections. It is extremely unlikely, however, that fibers of passage account for the entirety of the anterograde and retrograde labeling results. First, anterogradely labeled axons would have to originate from an input source that enters at all edges of the Imc and then sends axons across the entirety of the rostrocaudal and dorsoventral axes of the nucleus. Second, retrogradely labeled somata would have to be Imc neurons that project through the length of the Imc without expressing any axonal boutons within the Imc. Both of these two unlikely scenarios would have to be true to invalidate our conclusions. Instead, the presence of global intranuclear connectivity is a much more parsimonious interpretation and is completely consistent with the physiological results showing that inhibitory currents can be evoked by activating Imc neurons that are distant from a recorded Imc neuron.

The finding of reciprocal inhibitory connectivity within the Imc provides a crucial link between two previous lines of study: (1) experimental work that required the action of a reciprocal, global inhibitory network in the midbrain [13], and (2) theoretical work that established direct inhibition of inhibition as the most efficient and reliable motif for implementing feedback inhibition to permit flexible selection in dynamic environments [2]. With the recent finding that the Imc does, indeed, mediate global competitive suppression in the OT [6], we predict that focal blockade of inhibition within the Imc will reduce the flexibility of stimulus selection in the OT.

### Competitive feedback inhibition versus conventional inhibition

Imc neurons exhibit distinctive anatomical and functional properties that support their specialized role in stimulus selection in the OT. Imc neurons receive a topographically organized input from the OT and send projections broadly across the space maps of the OT and the neighboring, cholinergic nucleus, Ipc [7]. This broad inhibitory projection pattern, emanating from the Imc, enables activity in one portion of the OT space map to inhibit activity in all other portions, thereby aiding competitive stimulus [6] selection.

The pattern of extensive inhibitory connectivity within the Imc, revealed in this study, adds another computational component to stimulus selection. Models show that this architecture enables the efficient and reliable adjustment of selection boundaries based on the relative properties of the competing stimuli. Consistent with these models, previous work demonstrates that the OT and the Ipc, both of which receive direct input from the Imc, adjust their selection boundaries flexibly depending on the relative strengths of competing stimuli [5], [14]. However, while the reciprocal inhibitory connectivity within the Imc provides the most rapid and efficient source of feedback inhibition for flexible selection, alternate, indirect pathways through the OT and Ipc may also contribute to selection.

The pattern of global connectivity in the Imc reported here contrasts with conventional feedforward inhibition, which occurs ubiquitously in the brain [15]. Conventional inhibition is local [10] and is often proportionately “in balance” with excitatory drive, such that stimuli that evoke strong excitation also evoke strong, similarly tuned inhibition [16]. The spatial extent of this inhibition is typically slightly broader than that of excitation, with both exhibiting decaying strengths as the distance from the center of the receptive field increases, resulting in a classical ”Mexican hat” spatial profile. Such inhibition is crucial for shaping neural responses to single stimuli or to multiple, nearby stimuli in various cortical and sub-cortical areas, and can serve a variety of computational functions, including feature analysis, gain control and circuit stabilization [15].

The pattern of connectivity in the Imc establishes direct feedback between inhibitory neurons representing distinct, distant spatial locations, and supports a different computation: it enables the comparison of representations across the entire space map for the purposes of stimulus selection. Unlike in conventional circuits, the spatial topography of inhibition in the Imc is not matched to the topography of excitation: whereas Imc neurons have spatially restricted, excitatory receptive fields [17], we show that potent inhibitory drive can originate from anywhere in the nucleus. Furthermore, unlike conventional, feedforward inhibition, the strength of lateral inhibition within the Imc is independent of distance (Fig. 2J, K). Thus, the connectivity within the Imc supports a fundamentally different computation from that supported by local, center-surround inhibition that operates in many brain structures, including the neocortex.

Various studies have revealed the existence of long-range inhibition across species [18], [19] and in several brain areas: Area RA of the bird song system [20], hippocampal hub neurons [21], [22], between regions of the neocortex [23], [24], as well as between entorhinal and perirhinal cortex and hippocampus [23]–[26]. Furthermore, the function of long-range inhibition may be dynamically controlled: recent work indicates that in the awake mouse neocortex, long-range inhibition is modulated by anesthesia [27]. Thus, specialized, long-range inhibitory networks are present in the forebrain as well, and may be utilized for global, competitive selection.

### Reciprocal inhibition of inhibition in other brain areas

The motif of reciprocal inhibition of inhibition has been reported in other brain structures across species: neocortex [15], [28], hippocampal formation [25], [29], thalamic reticular nucleus (TRN) [30], and cerebellum [31]. A difference between the reciprocal connectivity within the Imc and that within the majority of these areas is that of spatial extent: unlike intra-Imc inhibition, reciprocal inhibitory connections in most other structures are far less widespread, rarely extending across more than a small portion of the structure [30]–[32], but see [25].

Spatially local, reciprocal inhibition has been proposed to mediate a variety of functions. For instance, in the mammalian neocortex and hippocampus, reciprocal inhibition is hypothesized to be crucial for synchrony between inhibitory basket cells to generate gamma oscillations in a local circuit [28]; in the TRN, this motif plays an important role in desynchronizing the TRN-thalamic network to avoid the generation of pathological oscillations [33]; and in the cerebellum, reciprocal inhibition between Golgi cells may act to restrict feedback excitation [31]. In these areas, it is possible that, despite the relatively restricted spatial extent of this motif, the motif could be operating “globally” across a full range of values of a feature that is represented locally within the structure.

The pattern of inhibition within the Imc is clearly global, with functional implications as revealed by both empirical evidence and modeling: global intra-Imc inhibition would support competitive stimulus selection and would improve the speed and reliability of flexible selection and sharpen selection boundaries [2]. This motif of global inhibition of inhibition may be expected where neural computations demand flexibility, speed, reliability, or strongly nonlinear processing. Such computations are required for a broad range of critical functions, including perception, attention, and decision-making. Future experiments will be required to test whether this motif is, indeed, implemented to accomplish these critical functions.

## Materials and Methods

### *In vitro* slice preparation

This study was carried out in strict accordance with the recommendations of the Guide for the Care and Use of Laboratory Animals of the National Institutes of Health. The protocol was approved by the Administrative Panel on Laboratory Animal Care at Stanford University (Protocol Number: 11800). All manipulations were performed under isoflurane anesthesia, and all efforts were made to minimize suffering. Transverse and horizontal slices (300 µm) were prepared from White Leghorn chicks (*Gallus gallus*), aged p0–p2, as previously described [8]. At these ages, these precocial birds are self-sufficient and have refined visual capabilities, including the abilities to imprint on a parent and to discriminate small food pellets from non-food stimuli. These ages were selected because they maximize the visibility of Imc neurons; with age, there is progressive myelination, and tissue opacity increases dramatically.

### *In vitro* slice recordings

Whole-cell and cell-attached patch recordings were performed in a submerged chamber at room temperature. 2–4 MΩ pipets were filled with (concentrations in mM), Cs Gluconate (130); CsCl (10); NaCl (2); HEPES (10); EGTA (4). Data were amplified using a Multiclamp 700B (MDC) and digitized by a Digidata 1322 (MDC) at a sampling rate of 10 kHz and acquired using pClamp 9/10. Neurons with series resistance <25 MΩ that did not fluctuate (<25%) were used for analysis. Series resistance was not compensated, but V_m_ was corrected for a −16 mV junction potential at the time of the recording. Thus, to hold the neuron near 0 mV, the voltage command was +15 mV.

100 µM MNI-glutamate (Tocris, 1490) was added to a recirculating solution of 20–25 mL ACSF. Somata were visualized and patched using a 63× Achroplan objective and DODT illumination. Upon successful break-in, the objective was switched to a 5× Fluar objective for photostimulation. A 355 nm laser (DPSS 3501) was directed through the microscope (Axioskop, Zeiss) using a custom setup of galvanometer-controlled mirrors, controlled by custom software. Laser pulse duration was between 100–200 µs. Median spot size, measured by shining the laser spot onto a piece of paper under the objective, was 85 µm. Large extents of the Imc were assayed using sequential scan areas.

In 3 experiments, 10 µM GABAzine (Tocris, 1262) was added to the MNI-glutamate solution.

### Anatomy

#### In vivo virus injection

A p13 chick was anesthetized with isoflurane and prepared for surgery. 250 nL of A3V driving eGFP under the RSV promotor (generously provided by D. Watanabe [9]) virus was injected into the Imc over a period of 2.5 minutes. Following completion of injection, the needle was left in the brain for 5 minutes. The needle was extracted slowly, the scalp was sutured, and the animal was monitored postoperatively.

After 19 days survival time, the animal was anesthetized deeply and transcardially perfused with cold saline and 4% paraformaldehyde. After an overnight postfixation, the brain was sunk in 30% sucrose and sectioned in the transverse plane to generate 40 µm sections.

Non-immunostained sections were mounted and coverslipped, and imaged on an epifluorescent microscope.

For immunodetection of parvalbumin, sections were placed in 5% normal goat serum in PBST (“block solution”) for 1 hour. Mouse anti-parvalbumin (Sigma, P3088): 1/2000 was diluted in the block solution and applied to sections for 2 nights at 4°C. Secondary antibodies were added at 1/300 to the block solution and applied to sections for 2 hours. Images were acquired using a Zeiss LSM 510 confocal microscope at 20× and 63×. 20× images were taken with a Z separation of 1 µm. Z-stacks were compressed to a maximum projection using Fiji image analysis software.

#### In vitro dye injection

Horizontal slices were prepared and placed into a static interface chamber. 10% fluororuby was dissolved in PBS+0.1% triton-X and pressure injected into the Imc using a pipette with 25–40 µm diameter bore. Slices were incubated for >4 hours. Slices were fixed in 4% paraformaldehyde overnight, sunk in 30% sucrose, resectioned at 40 µm, and coverslipped.

### Analysis

#### Physiology

Analysis of recordings was performed with custom software in Matlab (Mathworks, Natick, MA). Locations of photostimulation sites were matched to recorded traces. Inward currents recorded at −60 mV that 1) had peaks greater than 250 pA below baseline, and 2) occurred 3–5 ms following the laser pulse were considered to be direct. Outward currents recorded at 0 mV that 1) had peaks greater than 5 pA above baseline, and 2) occurred 4–20 ms following the laser pulse were considered to be evoked. Heat-maps of current amplitude as a function of the photostimulation location were generated using the ‘jet’ colormap in Matlab.

Distances from the photostimulation site to the recorded neuron were calculated in two ways. For all recordings in the transverse plane and MNI-C recordings in the horizontal plane, linear distance was calculated using a Euclidean distance: distance = √((x_photostim site_−x_cell_)^2^+(y_photostim site_−y_cell_)^2^). IPSC recordings in the horizontal plane underwent a modified analysis. Due to the exceptionally large distances between recording and stimulation sites, the curvature of the Imc introduced a potential source of error (see Fig. 4A). To calculate distances, the photostimulation maps were straightened, using the Straighten plugin in ImageJ (http://rsbweb.nih.gov/ij/plugins/straighten.html), and the separations between the photostimulation site and neuron were measured as distances along the straightened axis of the Imc.

#### Anatomy

Analysis of pixel intensity was performed by obtaining a maximal projection from a confocal stack (20× image). Using Matlab, a 10×10 pixel region of interest was manually selected for each soma. The average intensity was calculated for that region of interest. Analysis of cell distance was performed by utilizing the X-Y coordinates of the previously mentioned, manually selected somata. A nearest-neighbor analysis was performed calculating the Euclidean distance between a given soma and all other selected somata in the image. The shortest distance (i.e. distance to the nearest neighbor) for each cell was computed for all somata in the image: small values indicated that each cell had nearby neighbors; large values indicated distant neighbors.
